# Time to recovery and its predictors among pediatric patients with severe community-acquired pneumonia admitted to public general hospitals in Tigray, Ethiopia, 2025: A prospective follow-up study

**DOI:** 10.1371/journal.pone.0358705

**Published:** 2026-09-24

**Authors:** Tekle Gebremeskel Ygzaw, Teklewoini Mariye Zemicheal, Gebreamlak Gebremedhn Gebremeskel, Teklebrhan Welderufael Kidane, Berhe Gebrehiwot Tewele, Ngsti Gebremicheal Beyene, Guesh Teklu Woldemariam, Nebiat Desale Gidey, Gebremedhn Sibhat Weldegebrieal, Geberziher Geberslassie Weleargay, Birhanu Kassie Reta, Binyam Gebrehiwet Tesfay

**Affiliations:** 1 Araya Kahsu Health Science Colleges, Aksum, Ethiopia; 2 Aksum University College of Health Sciences, Aksum, Ethiopia; 3 Adigrat University College of Health Sciences, Adigrat, Ethiopia; 4 Aksum St. Mary General Hospital, Aksum, Ethiopia; Menzies School of Health Research: Charles Darwin University, AUSTRALIA

## Abstract

**Background:**

Globally, pneumonia is a major cause of illness and death in children, with over 1,400 cases per 100,000 children each year. In sub-Saharan Africa, pneumonia is responsible for approximately 172 deaths per 1,000 live births. Ethiopia has the fifth highest mortality rate from pneumonia among children under five, especially severe community-acquired pneumonia. In Ethiopia, there are limited prospective follow-up studies on time to recovery and its predictors among pediatric patients with severe community-acquired pneumonia. However, no prospective follow-up study conducted on time to recovery and its predictors among pediatric patients with severe community-acquired pneumonia admitted to public general hospitals of Tigray.

**Methods:**

A prospective follow-up study was conducted among 305 children using consecutive sampling to select the study participants from December 20, 2024, to February 20, 2025. The collected data were entered into Epi-Data version 4.7 and then exported to STATA version 14 for further statistical analysis. The Kaplan–Meier curve was used to estimate recovery time. Both bivariable and multivariable Cox regression models were applied to analyze the data. Finally, the results were reported with 95% confidence intervals, and variables with a p-value < 0.05 were considered statistically significant.

**Result:**

The recovery rate among pediatric patients was 85.2%, with a median recovery time of 5 days. The overall incidence of recovery rate was 16.30 per 100 person days observation (95% CI: 14.43–18.40). Being rural residence (AHR = 0.69;95%CI (0.53–0.90), late presenter to seeking care (AHR = 0.56; 95% CI (0.35–0.88), absence of danger sign (AHR = 1.48; 95% CI: 1.07–2.05)), absence of comorbidity (AHR = 3.02; 95% CI: 2.15–4.22) and not receiving oxygen therapy (AHR = 1.71; 95% CI: 1.20–2.41), were significant predictors.

**Conclusions:**

The median recovery time of patients was considerably high. Rural residence, late presentation for care, absence of danger signs, absence of comorbidity, and not receiving oxygen therapy were statistically significant predictors of recovery time.

## Introduction

According to the WHO, community-acquired pneumonia (CAP) in children is a lung infection that develops outside a hospital. Children with CAP typically present with coughing, breathing difficulties, rapid breathing rates, chest indrawing, and are diagnosed within 48 hours of hospitalization [1,2].

Severe community-acquired pneumonia (SCAP) can be defined as CAP plus at least one of the following: Oxygen saturation <90%, general danger signs, severe respiratory distress, central cyanosis; or stridor in a calm child, increased respiratory rate (RR > 50 b/ minute) in children aged two months to 12 months, and RR > 40 b/ minute in children aged 12 months to 59 months, RR > 30/ min in children aged 60 months and above [3,4].

Globally, pneumonia is a major health problem for children, causing between 150 and 156 million new cases and leads to hospitalization for 10–20 million children annually [5]. According to the UNICEF report of 2023, Globally there are over 1,400 cases of pneumonia per 100,000 children, or 1 case per 71 children every year, with the highest rates in low and middle-income countries such as South Asia 2,500 cases per 100,000 children and West and Central Africa 1,620 cases per 100,000 children [6].

Africa has the highest rate of pneumonia in children under five, with approximately 35 million cases occurring each year [7]. In sub-Saharan Africa, pneumonia is a major health problem for children, causing approximately 172 deaths per 1,000 live births [8]. Pneumonia was common in East Africa, with a prevalence of 34% [9].

In Ethiopia, approximately 3,370,000 children under the age of five develop pneumonia each year, resulting in an estimated 32,000 pneumonia-related deaths annually [10,11]. Furthermore, approximately 16.09% of deaths among children under five were attributed to SCAP [12]. Additionally, 28% of children under five admitted to the hospital with SCAP died during the follow-up period [13].

Prolonged hospitalization for children with SCAP imposes a substantial financial burden on both developed and developing countries, particularly in resource-limited settings [14,15]. Although mortality is a common indicator of disease severity, prolonged hospital stay is also an important measure of the severity and clinical burden of SCAP [16]. According to the United States Centers for Disease Control and Prevention (CDC), children younger than 15 years who are hospitalized with pneumonia typically have a hospital stay of 5 days. Therefore, a hospital stay exceeding 5 days is considered prolonged [8].

Extended hospital stays for children with SCAP can lead to significant emotional and psychological challenges for both the affected children and their families [17,18]. The adverse effects of prolonged hospitalization extend beyond the child, placing substantial emotional, social, and economic burdens on mothers, caregivers, families, society, and the nation as a whole. In addition, families experience considerable financial hardship due to hospitalization costs, with the duration of hospitalization accounting for 46.8% of the total household expenditure for a single episode of SCAP [19]. Furthermore, the cost of hospitalization for severe pneumonia, particularly SCAP, ranges from 26.6% to 115.8% of a family’s monthly income [20].

According to different studies, several factors can impact recovery time for children with SCAP. These factors include socio-demographic characteristics (like age and rural residence), nutritional status, immunization status, presence of danger signs, comorbidities, delayed care-seeking, and the type of medication used [1,14,21–24].

Ethiopia has been working on and implementing initiatives under its Health Sector Transformation Plans to reduce pneumonia rates by 2025 and 2035. These plans aim to decrease the child mortality rate to 31 per 1,000 live births by 2025 and to 14 per 1,000 live births by 2035 [25].

The government has implemented several strategies to reduce childhood SCAP, including integrated community case management (ICCM), the integrated management of newborn and child illnesses (IMNCI) program, pneumococcal conjugate vaccine (PCV) and Haemophilus influenzae type b (Hib) vaccinations, nutrition programs, the Health Extension Program, Water, Sanitation, and Hygiene initiatives and improvements in healthcare infrastructure. Despite these efforts, SCAP remains a major health challenge, with the country ranking among the top five globally for the highest child mortality due to pneumonia, at 62 deaths per 1,000 children [8,11,26]. Furthermore, it is associated with higher hospitalization rates and lower recovery rates [27]. Still, pneumonia continues to be prevalent, affecting 20.68% of children under five in Ethiopia [28].

SCAP remains a major cause of morbidity and mortality among children. In the post-conflict setting of Tigray, damage to health facilities, shortages of essential medicines and medical supplies, and disrupted healthcare services may delay recovery and prolong hospitalization among children with SCAP. However, evidence on the time to recovery and its predictors in this setting is limited. Therefore, this study aims to assess the time to recovery and its predictors among pediatric patients with SCAP admitted to public general hospitals in Tigray.

## Methods and materials

### Study area and period

The study was conducted in public general hospitals in Tigray, Ethiopia. Tigray is located in the northern part of the country. The region has seven administrative zones and 14 public general hospitals. The study areas were Aksum St. Mary Hospital, Shire Suhul Hospital, Adigrat Hospital, Mekelle Hospital, Miyani Sheraro Hospital, and Abiy Adi Hospital. Each of these hospitals has a pediatric ward that provides services to pediatric patients. The study was conducted from December 20, 2024, to February 20, 2025.

### Study design

Hospital-based prospective follow up study was conducted.

### Source population

All children with SCAP who were admitted to the pediatric ward of public general hospitals of Tigray.

### Study population

All children with SCAP who were admitted to the pediatric wards at selected public general hospitals of Tigray during the data collection period.

### Inclusion criteria and exclusion criteria

#### Inclusion criteria.

All Children aged above 2 months to under 15 years with SCAP who were admitted on pediatric ward to the selected public general hospitals of Tigray during the study period were included.

#### Exclusion criteria.

Children whose parents or guardians do not provide informed consent for their participation in the study are excluded

### Sample size determination

The sample size was calculated using STATA version 14. The calculation was based on the assumptions of 5% types I error, 80% power, and a standard deviation of 0.5. The probability of an event was 0.8956, based on a previous study conducted at Asella Referral and Teaching Hospital in Oromia, Ethiopia, with a hazard ratio of 1.55 for the predictor of the child’s age [22].The estimated sample size was 183. After adding a 10% non-response rate and applying a design effect of 1.5, the final sample size for this study was 305.

### Sampling procedure and technique

The study hospitals were selected using a simple random sampling (lottery) method. Based on admissions during the previous two months, 424 children with SCAP were admitted to the pediatric wards of the six selected public general hospitals (Aksum St. Mary, Shire Suhul, Adigrat, Mekelle, Miyani Sheraro, and Abiy Adi Hospitals). The sample was proportionally allocated to each hospital (71, 78, 46, 55, 30, and 25, respectively), yielding a total sample size of 305. A consecutive sampling (consecutive recruitment) method was used. Eligible children were recruited consecutively during the study period as they were admitted and met the predefined eligibility criteria. Recruitment continued until the required sample size of 305 participants was reached, and all eligible children admitted thereafter were not included in the study.

### Study variables

#### Dependent variable.

Time to recovery from SCAP.

#### Independent variables.

**Socio-demographic factors** include: Age of children, sex, residences, Education of care giver, Occupation of care giver

**Vaccination status factors** include: Fully vaccinated, partially vaccinated and not vaccinated

**Nutritional status factors** include: Exclusive breast feeding, mal-nutrition

**Clinical-related factors** include: The presence of comorbid disease, duration to seeking care, presence of danger sign and IV antibiotic given

### Operational definitions

**Recovery time**: Defined as the time from the date of admission to the event or recovery from SCAP during the study period [1,22]

**Follow-up time**: The time of admission to the pediatric ward from SCAP until either an event or censorship occurs [22].

**Recovery**: Children improved from SCAP as declared by the clinician/physician [1].

**Censored**: Children who were defaulter,referred, transferred, or died before recovering from SCAP [1].

**Time origin**: The time of admission of children in to pediatric ward from SCAP [22].

**Duration to seek care**: Duration in days from onset of symptoms to health facility visit. Early presentation defined as seeking medical care with in the first 5days of illness. Late presentation: defined as to seeking medical care after 5days of illness [1,29].

**Danger signs**: If the child has loss of consciousness, abnormal body movement, vomiting of everything, convulsions, and inability to feed in addition to SCAP [1,3].

**Co-morbidity**: is a child has any disease condition (acute or chronic) present at admission in addition to SCAP which includes hyperactive airway disease, severe acute malnutrition, congestive heart failure, rickets, down syndrome, retroviral infection, tuberculosis, acute gastroenteritis, pertussis, anemia, meningitis, measles, bronchitis, heart failure and others [1].

**Immunization status: Full vaccinated status** was children who had completed all forms of vaccinations; **partially vaccinated** children who had taken at least 1 dose of PCV and **non-vaccinated** children who had never vaccinated PCV and other vaccines [1,3,25]

**Moderate acute malnutrition**: Z-scores of between −3SD and −2SD [30].

**Severe acute malnutrition**: Z-scores of <−3SD and the presence of bilateral pitting edema [30,31]

### Data collection tools and procedures

The questionnaire was adapted by reviewing from previous similar literature [1,14,21,23,24,32–35]. Data was collect using an interviewer-administered questionnaire with direct face-to-face interviews with the mothers or care givers. Document reviews were also considered. The questionnaire was including three sections: socio-demographic variables, nutritional and immunization status, and clinical-related variables.

Data was collected by interviewing the caregivers of the child and by reviewing daily patient records by one trained health professional (BSc in Nursing) and one BSc Nurse assigned as a supervisor for each hospital. Data collectors and the study supervisor was train on the content of the questionnaire and the data collection procedure.

### Data quality control

The questionnaire was initially prepared in English then translated into the local language in Tigrigna. The questionnaire was pretest in a similar setting by the research investigators prior to the data collection on 5% of the total sample size at Adwa general hospitals that who were not selected in the study. Revisions and adjustments were performed after the pre-test. The mothers of each child were orientating verbally about the purpose and usefulness of the study. The collected data were check on each day of activity for consistency and completeness by the immediate supervisors and principal investigator. The data collectors and supervisor were trained. Furthermore, the data collection questionnaire and all data collection processes were ensured, check, and supervised for content and completeness.

### Data analysis

After ensuring the data was complete and accurate, it was entered into Epi-Data version 4.7 and then exported to STATA version 14 for analysis. Missing values were addressed before analysis. Descriptive analyses (percentages, median, IQR) were used to describe the frequency and percentage of the dependent and independent variables. To classify children’s nutritional status, were compared to WHO child growth standards. Additionally, the life table were used to estimate cumulative survival probabilities after admission. Kaplan Meier survival curve and log-rank test were used to describe the survival function of the time to recovery of pediatric patients with SCAP. Furthermore, both bi-variable and multivariable Cox regression models were used. Based on bivariable analysis, those variables having a p-value < 0.25 in the bivariable analysis was transferred to the multivariable analysis. The Crude Hazard Ratio (CHR) and Adjusted Hazard Ratio (AHR) with 95% confidence interval were used to measure the strength of association between the independent and dependent variables and the P-value ≤ 0.05 were considered statistically significant. The Cox proportional hazard regression assumption was tested by using the Schoenfeld residual test. Moreover, the overall model fitness was checked graphically by using the Cox Snell residual. Finally, the findings were presented in text, tables, and figures using frequencies and summary statistics.

### Ethical clearance

An official ethical clearance letter was obtained from the Aksum University College of Health Sciences Institutional Review Board (IRB) on 03 December 2024, with Protocol of Approval AKU-IRB 084/2024. A formal letter was issued by Aksum University to the Tigray Regional Health Bureau, which subsequently issued permission to the medical directors of the selected public general hospitals. Following ethical approval, communication was established with the medical directors and heads of the pediatric wards at each selected hospital.

Written consent was obtained from the parents or guardians of all children admitted with SCAP after providing a clear explanation of the study’s purpose, procedures, and potential risks. All information collected was kept confidential and securely stored. Participants’ names and other identifying information were not recorded on the questionnaires. All measurements and data collection procedures were conducted without causing harm to the children and access to the study data was restricted to the principal investigator. All methods and procedures were conducted in accordance with the principles of the Declaration of Helsinki.

## Results

### Socio demographic variables

A total of 305 pediatric patients with the diagnosis of SCAP were followed. Out of 305 pediatric patients, 188(61.64%) were in the age group between 1 and 5 years, 178 (58.36%) of them were males, and 165 (54.10%) of the children were living in urban areas (Table 1).

**Table 1:** Socio demographic variables of pediatrics patients and caregivers with SCAP in selected public general hospitals of Tigray, Ethiopia, 2025 (n = 305).

Variables	Categories	Recovery	Censored	Total(n = 305)
Age	<1year	88(28.85)	19(6.23)	107(35.08)
	1-5year	165(54.10)	23(7.54)	188(61.64)
	5-15year	7(2.30)	3(0.98)	10(3.28)
Sex	Male	146(47.87)	32(10.49)	178(58.36)
	Female	114(37.38)	13(4.26)	127(41.64)
Place of residence	Urban	148(48.38)	17(5.57)	165(54.10)
	Rural	112(36.72)	28(9.18)	140(45.90)
Educational status of caregiver	Not able to read and write	37(12.13)	7(2.30)	44(14.43)
	Able to read and write	55(18.03)	14(4.59)	69(22.62)
	Grade 1–8	74(24.26)	15(4.92)	89(29.18)
	Grade 9–12	65(21.31)	7(2.30)	72(23.61)
	Diploma and above	29(9.51)	2(0.66)	31(10.16)
Occupation of caregivers	House wife	90(29.51)	20(6.56)	110(36.07)
	Farmer	48(15.74)	11(3.61)	59(19.34)
	Daily laborer	32(10.49)	5(1.64)	37(12.13)
	Government employee	26(8.52)	2(0.66)	28(9.18)
	Marchant	21(6.89)	1(0.33)	22(7.21)
	Others	43(14.10)	6(1.97)	49(16.07)

### Nutritional and vaccination status of patients admitted with SCAP

Two hundred fifty-two (82.62%) were exclusively breastfed. Among all study participants, 59 (19.34%) were underweight, 39 (12.79%) had stunting, and 43 (14.10%) had wasting. Regarding vaccination status, 176 (57.70%) were fully vaccinated. Furthermore, a significant majority, 272 (89.18%), of the participants were exposed to sunlight (Table 2).

**Table 2:** Nutritional and vaccination status of pediatrics patients with SCAP in selected public general hospitals of Tigray, Ethiopia, 2025 (n = 305).

Variables	Categories	Recovery	Censored	Total (n = 305)
Exclusively breast feed	Yes	219(71.8)	33(10.82)	252(82.62)
	No	41(13.44)	12(3.93)	53(17.38)
Starting of complementary feeding	<6month	43(14.10)	9(2.95)	52(17.05)
	>6 month	197(64.6)	31(10.16)	228(74.75)
	Not applied	20(6.56)	5(1.40)	25(8.20)
Vaccination status	Fully vaccinated	152(49.83)	24(7.87)	176(57.70)
	Partially vaccinated	108(35.41)	21(6.89)	129(42.30)
Weight for age	Normal	209(68.53)	37(12.13)	246(80.66)
	Underweight	51(16.72)	8(2.62)	59(19.34)
Height for age	Normal	228(74.75)	38(12.46)	266(87.21)
	Stunting	32(10.49)	7(2.30)	39(12.79)
Weight for Height	Normal	224(73.44)	38(12.46)	262(85.90)
	Wasting	36(11.80)	7(2.30)	43(14.10)
Sunlight exposure	Yes	233(76.39)	39(12.79)	272(89.18)
	No	27(8.85)	6(1.97)	33(10.82)

#### Clinical related information variables of patients admitted with SCAP.

Two hundred sixty-three (86.23%) were children who presented early for care, while 73 (23.93%) had a history of ARTI. Regarding body temperature at admission, 160 (52.46%) of the children had a high-grade fever which is greater than 37.5^o.c^. About 76 (24.92%) of the children had danger signs at admission. Additionally, 118 (38.7%) of the children had comorbidities on admission. In terms of treatment, 192(62.95) %) received ceftriaxone (Table 3).

**Table 3:** Clinical related information variables of pediatrics patients and caregivers with SCAP in selected public general hospitals of Tigray, Ethiopia, 2025 (n = 305).

Variables	Categories	Recovery	Censored	Total(n = 305)
Duration of seeking care	<5day (Early presenter)	229(75.08)	34(11.15)	263(86.23)
	>5day (late presenter)	31(10.16)	11(3.61)	42(13.77)
Body temperature at admission	36.5^o.c^-37.5^o.c^	30(9.84)	6(1.97)	36(11.80)
	<36.5^o.c^	95(31.15)	14(4.59)	109(35.74)
	≥37.5^o.c^	135(44.26)	25(8.20)	160(52.46)
Oxygen saturation at admission	<90	102(33.44)	16(5.25)	118(38.69)
	≥90	158(51.80)	29(9.51)	187(61.31)
Previous history of ARTI	Yes	62(20.33)	11(3.61)	73(23.93)
	No	198(64.92)	34(11.15)	232(76.07)
Danger sign at admission	Yes	58(19.02)	18(5.90)	76(24.92)
	No	202(66.23)	27(8.85)	229(75.08)
Oxygen therapy	Yes	153(50.16)	22(7.21)	175(57.38)
	No	107(35.08)	23(7.54)	130(42.62)
Comorbidity	Yes	91(29.84)	27(8.85)	118(38.69)
	No	169(55.41)	18(5.90)	187(61.31)
Types of IV-line antibiotics	Ceftriaxone	161(52.79)	31(10.16)	192(62.95)
	Ampicillin and Gentamicin	59(19.34)	8(2.62)	67(21.97)
	Vancomycin	40(13.12)	6(1.97)	46(15.08)

### Treatment outcome and incidence rate of recovery from SCAP

**Fig 1:**
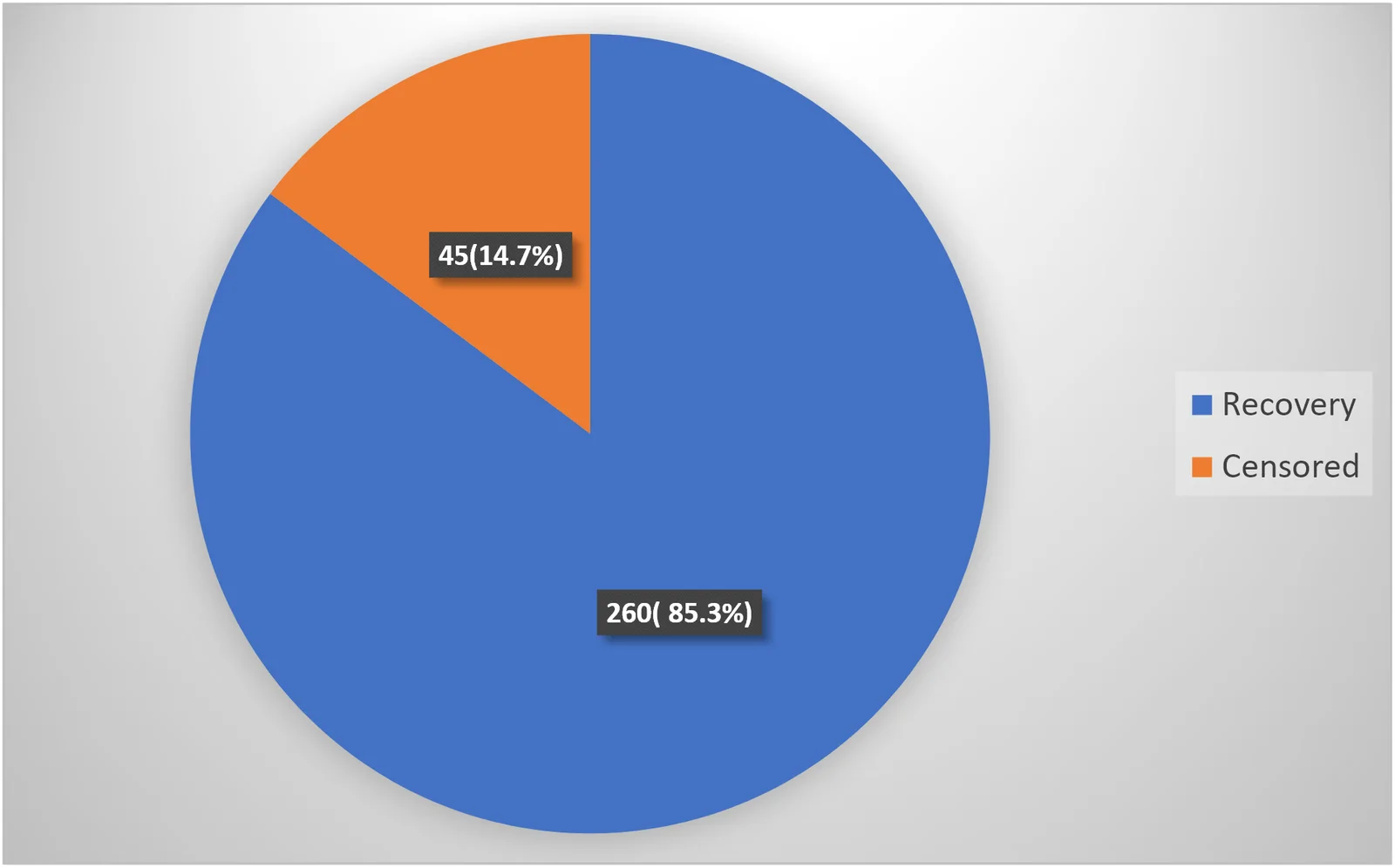
Treatment outcome of pediatrics patients with SCAP in selected public general hospitals of Tigray, Ethiopia, 2025 GC (n = 305).

Two hundred sixty (85.3%) of the pediatric patients recovered, while 45 (14.7%) were censored (Fig 1). Of the forty-five (45) censored children, 22 (7.2%) defaulted on treatment, 7 (2.3%) were transferred, 12 (3.9%) were referred, and 4 (1.3%) died. The follow-up duration for the children ranged from a minimum of 1 day to a maximum of 12 days, with a median recovery time was 5 days (95% CI:5–6). The total person-time risk was 1,595, with overall incidence rate of recovery was 16.30 per 100 person-days of observation (95% CI:14–18).

The cumulative probability of survival at 3^rd^, 6^th^, 9^th^ and 12 days were 85.50% (95% CI: 80.95 89.03%), 25.39% (95%CI: 20.29–30.78%), 4.91% (95%CI: 2.60–8.28%) and 0.29%% (95% CI: 0.01–2.58%) respectively (Table 4).

**Table 4:** Overall, of Life table of time to recovery pediatrics patients with SCAP in selected public general hospitals of Tigray, Ethiopia, 2025 (n = 305).

Time interval	Beginning total	Recovery	Censored	Survival	Std. Error	95% CI
1-2	305	0	2	1.0000	0.0000	
2-3	303	11	3	0.9635	0.0108	0.9351-0.9796
3-4	289	32	10	0.8550	0.0205	0.8095- 0.8903
4-5	247	44	8	0.7001	0.0270	0.6437 −0.7494
5-6	195	68	12	0.4482	0.0299	0.3889 - 0.5058
6-7	115	49	4	0.2539	0.0269	0.2029 - 0.3078
7-8	62	21	2	0.1665	0.0234	0.1235 - 0.2150
8-9	39	14	2	0.1051	0.0197	0.0705 - 0.1476
9-10	23	12	1	0.0491	0.0144	0.0260 - 0.0828
10-11	10	6	0	0.0196	0.0095	0.0067 - 0.0457
11-12	4	1	0	0.0147	0.0083	0.0041 - 0.0339
12-13	2	1	1	0.0029	0.0041	0.0001 −0.0258

### Kaplan-Meir survival estimates for SCAP recovery time

**Fig 2:**
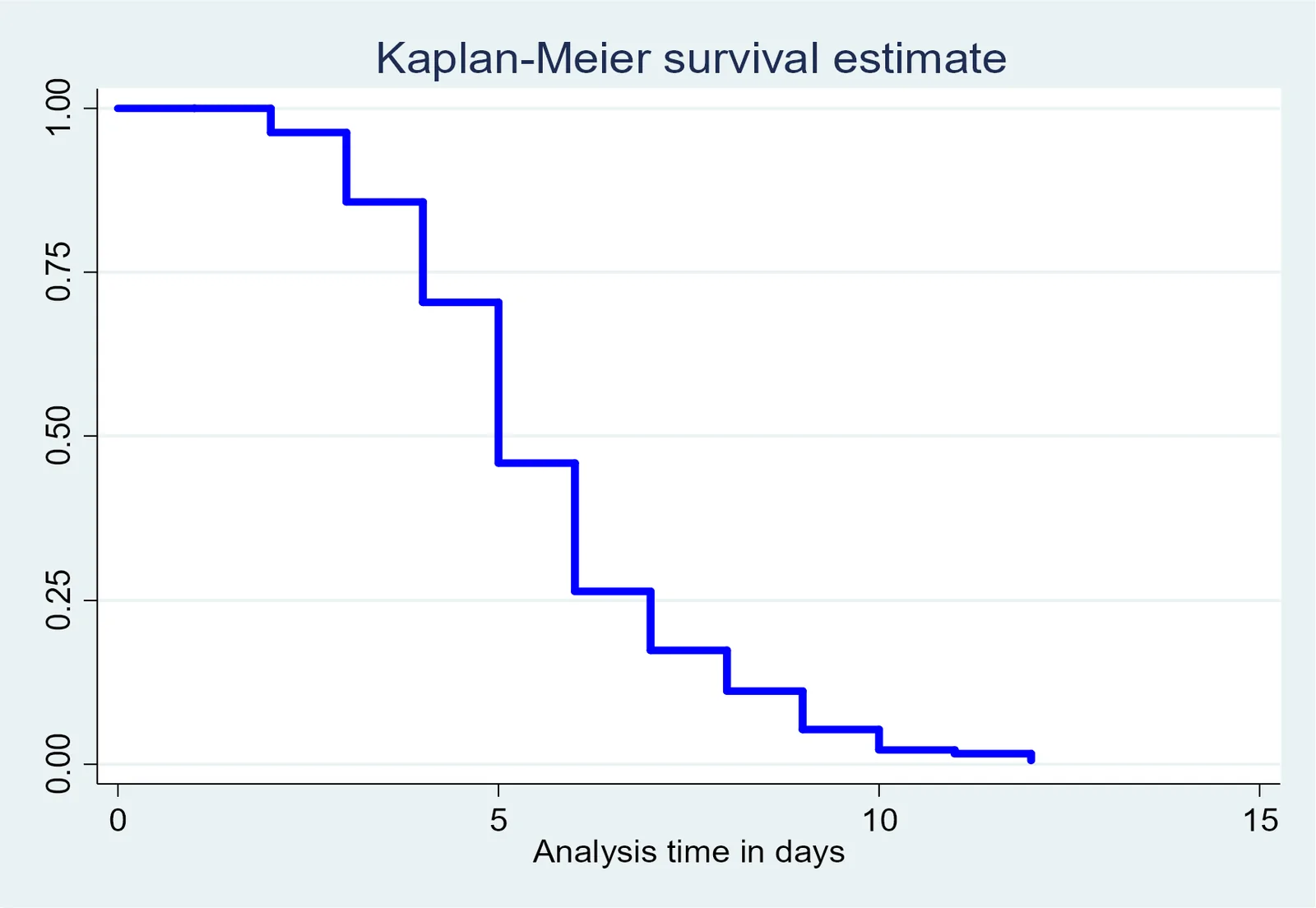
Over all Kaplan-Meir survival estimates of pediatrics patients with time to recovery from SCAP in selected public general hospitals of Tigray, Ethiopia, 2025 GC (n = 305).

Overall, the Kaplan-Meier survival estimate curve for SCAP shows that the median time to recovery was 5 days (95% CI:5–6). The Kaplan-Meier survival curve indicated that the curve tended to decrease rapidly within the first six days representing most children recovered from the disease within this time (Fig 2).

**Fig 3:**
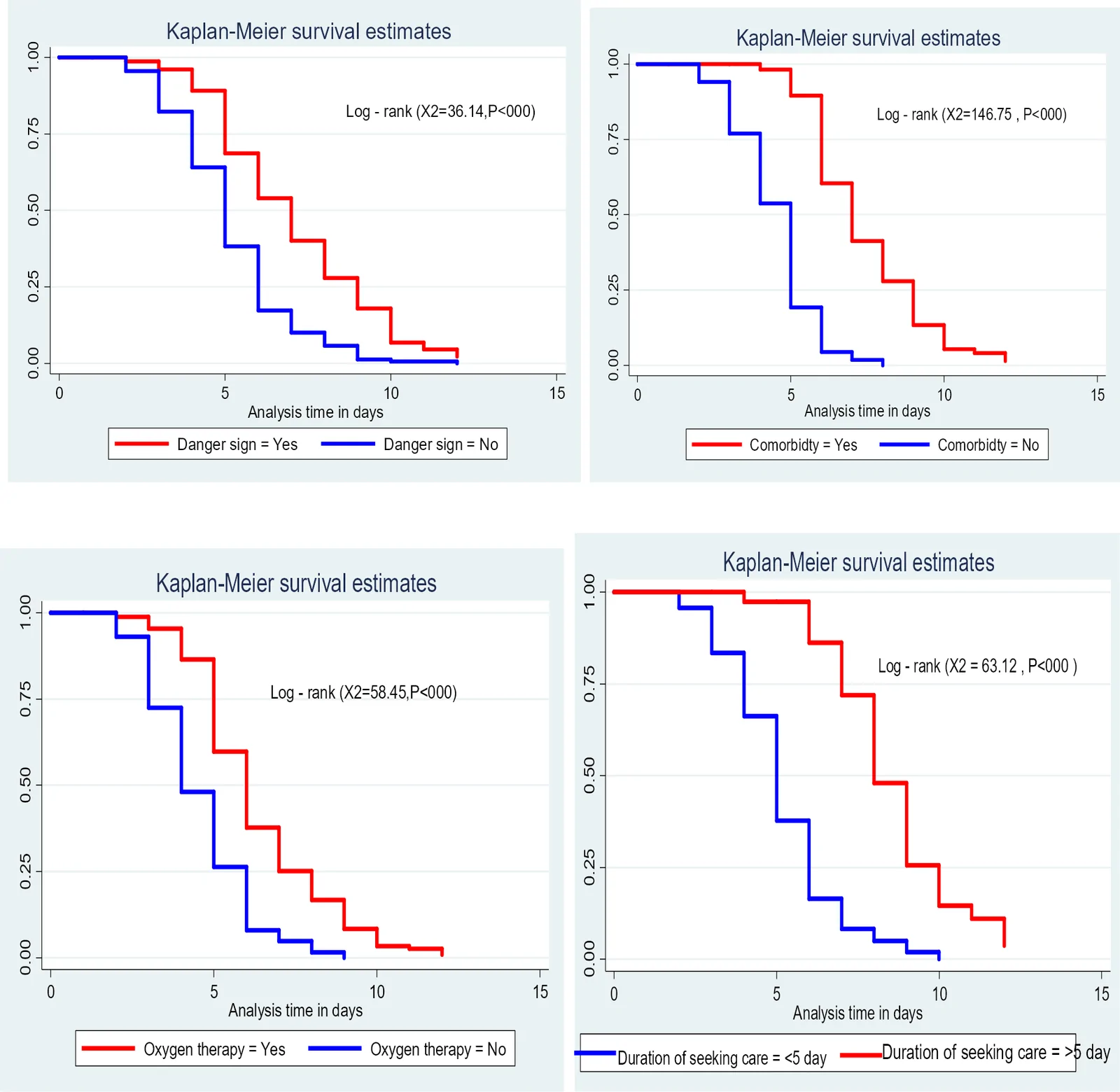
Kaplar Meier curve showing recovery time from SCAP in selected categorical variables in selected public general hospitals of Tigray, Ethiopia, 2025 GC (n = 305).

There was a statistically significant difference in recovery times among different groups of children. For instance, children with comorbidities at admission had longer recovery times compared to children without comorbidities. There was also a statistically significant difference in recovery time among different categories of oxygen therapy, duration to seeking care, and danger signs (Fig 3).

The median recovery time of children with SCAP differed across various categories of variables. For example, children from urban areas had a median recovery time of 5 days (IQR: 5–5), whereas children from rural areas had 6 days (IQR: 5–6). Similarly, the median recovery time among children with SCAP who had comorbidities was 7 days (IQR: 7–8), while those without comorbidities had a median recovery time of 5 days (IQR: 5–5). Furthermore, children who presented with danger signs at admission had a median recovery time of 7 days (IQR: 6–8), whereas those without danger signs had a median recovery time of 5 days (IQR: 5–5).

The log-rank test was performed to check for equality of survival time in the different predictors. Based on this test, survival time among different groups of predictors such as residence, weight for age, height for age, weight for height, exclusive breastfeeding, body temperature, oxygen saturation, duration to seeking care, danger signs, comorbidity, and oxygen therapy was significantly different in survival time at the 5% level of significance (Table 5).

**Table 5:** The median recovery time and comparisons of survival status between categories of predictors using log-rank test among pediatrics patients with SCAP in selected public general hospitals of Tigray, Ethiopia, 2025 (n = 305).

Variables	Categories	Median recovery time (95%CI)	Log- rank test X^2^ = Chi2	P- value
Residence	Urban	5(5−5)	27.10	0.0000
	Rural	6(5-6)		
Exclusive breast feeding	Yes	5(5−5)	8.77	0.0031
	No	6(6-7)		
Previous history of ARTI	Yes	6(5-7)	2.57	0.1088
	No	5(5−5)		
Oxygen saturation	< 90	6(6−6)	23.97	0.0001
	≥90	5(5−5)		
Body temperature	36.5^o.c^-37.5^o.c^	5(4-6)	18.03	0.0001
	<36.5^o.c^	5(4-5)		
	≥37.5^o.c^	6(5-6)		
WFA	Normal	5(5−5)	70.89	0.0000
	Underweight	8(7-9)		
HFA	Normal	5(5−5)	62.50	0.0000
	Stunting	9(8-9)		
WFH	Normal	5(5−5)	72.25	0.0000
	Wasting	9(8-9)		
Duration of seeking care	≤5 days	5(5−5)	63.12	0.0000
	>5 days	8(8-9)		
Danger sign	Yes	7(6-8)	36.14	0.0000
	No	5(5−5)		
Comorbidity	Yes	7(7-8)	146.75	0.0000
	No	5(5−5)		
Oxygen therapy	Yes	6(6−6)	58.45	0.0000
	No	4(4-5)		

Note: WFA = Weight for age, HFA = Height for age, WFH = Weight for height, ARTI = Acute respiratory tract infection: CI = confidence interval.

### Check proportional assumption cox hazard model

**Fig 4:**
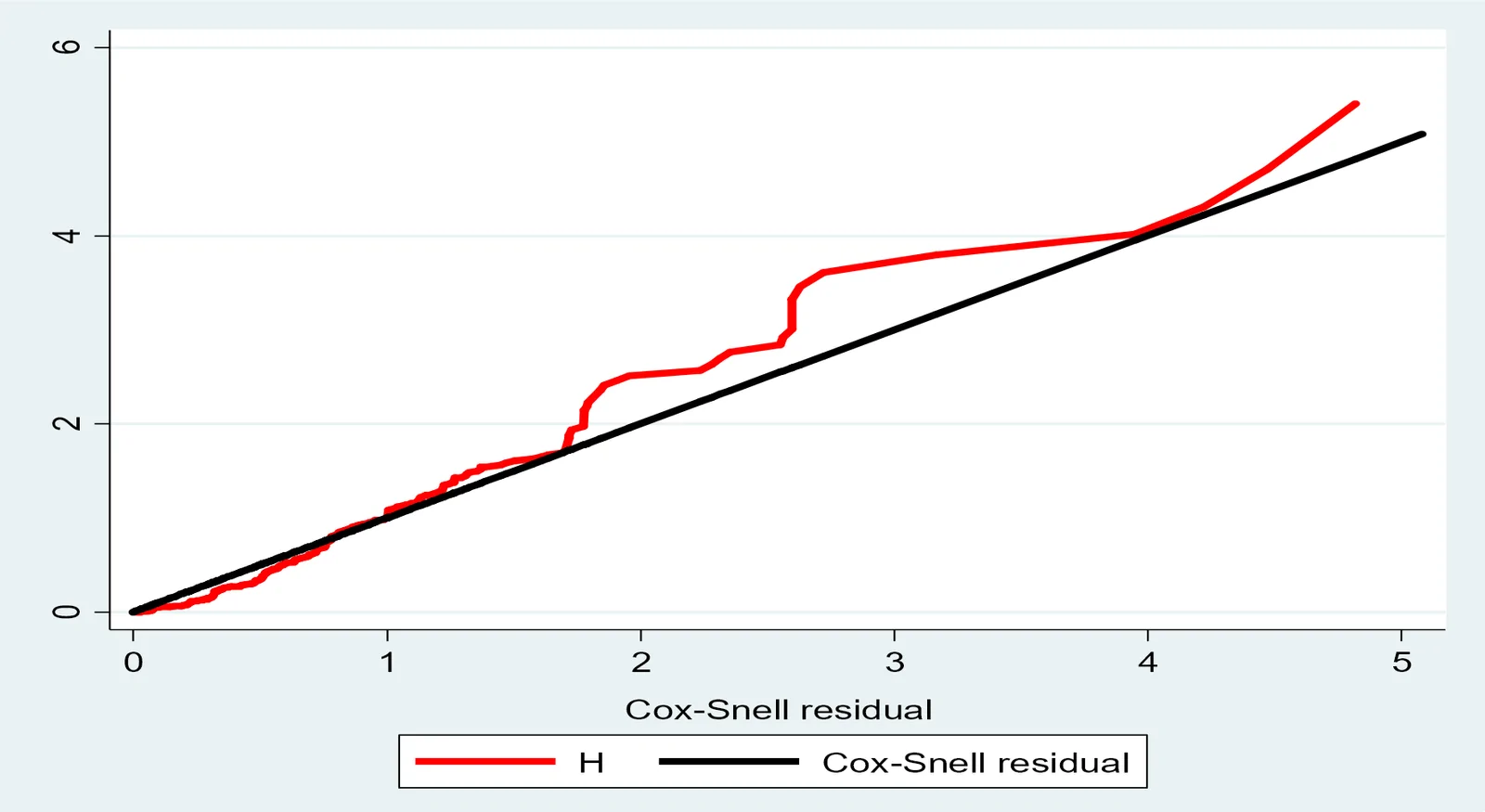
Cox-Snell Residual Graph for the goodness of model fitness that shows the hazard function follows the 45˚ closed to the baseline, in public general hospitals of Tigray, Ethiopia, 2025 GC (n = 305).

The Cox proportional hazards assumption was evaluated using the overall Schoenfeld residual global test, which indicated that the assumption was met (χ² = 18.01, P-value = 0.1155). All covariates satisfied the criteria for the proportional hazard assumption. Residuals were assessed using the goodness-of-fit test based on Cox-Snell residuals. The final model showed a strong fit to the data, as depicted in the figure below, where the hazard function closely follows the 45° line near the baseline (Fig 4).

### Predictors of recovery time from SCAP

Variables with a p-value <0.25 in the bivariable Cox proportional hazards model were included in the multivariable analysis. These variables included residence, exclusive breastfeeding, vaccination status, body temperature at admission, oxygen saturation at admission, weight-for-age, height-for-age, weight-for-height, duration of seeking care, presence of danger signs at admission, comorbidity at admission, and oxygen therapy. Finally, rural residence, late presentation in seeking care, absence of danger signs, absence of comorbidity, and not receiving oxygen therapy were found to be significantly associated with the time to recovery from SCAP**.**

Pediatric patients in rural areas had a 31% lower recovery rate from SCAP than those in urban areas (AHR = 0.69; 95% CI ((0.53–0.90). Pediatric patients who were late presentation in seeking care had a 44% lower recovery rate from SCAP than early presenters (AHR = 0.56; 95% CI (0.35–0.88). Pediatric patients without comorbidities had a 3.02 times faster recovery rate from SCAP than their counterparts (AHR = 3.02; 95% CI: 2.15–4.22). Pediatric patients without danger signs at admission had a 1.48 times higher recovery rate from SCAP compared to their counterparts (AHR = 1.48; 95% CI: 1.07–2.05). Additionally, Pediatric patients who did not receive oxygen therapy had a 1.71 times higher recovery rate from SCAP compared to those who received oxygen therapy (AHR = 1.71; 95% CI: 1.20–2.41) (Table 6).

**Table 6:** Bivariable and multivariable Cox proportional regression analysis time to recovery and its predictors among pediatric patients with SCAP admitted to selected public general hospitals of Tigray, Ethiopia,2025(n = 305).

Variables	Categories	Recovery	Censored	CHR (95% CI)	AHR (95% CI)	P- value
Residence	Urban	148	17	1	1	
	Rural	112	28	0.56(0.44-0.73) *	**0.69(0.53-0.90)** **	**0.007**
EBF	Yes	219	33	1.52(1.09- 2.13) *	1.12(0.79-1.60)	0.522
	No	41	12	1	1	
Previous history of ARTI	Yes	62	11	0.82(0.62-1.10) *	1.07(0.79-1.45)	0.645
	No	198	34	1	1	
Body temperature	36.5^o.c^-37.5^o.c^	30	6	1	1	
	<36.5^o.c^	95	14	1.20(0.79-1.80) *	1.00(0.65-1.54)	0.969
	≥37.5^o.c^	135	25	0.74(0.49-1.10) *	0.95(0.62-1.44)	0.815
Oxygen saturation	< 90	102	16	0.59(0.45-0.76) *	1.12(0.79-1.57)	0.511
	≥90	158	29	1	1	
Weight for age	Normal	209	37	1	1	
	Underweight	51	8	0.29(0.21-0.42) *	0.89(0.48-1.64)	0.717
Height for age	Normal	228	38	1		
	Stunting	32	7	0.25(0.17-0.39) *	0.81(0.41-1.58)	0.545
Weight for height	Normal	224	38	1	1	
	Wasting	36	7	0.24(0.16-0.36) *	0.57(0.28-1.14)	0.114
Duration of seeking care	≤5 days	229	34	1	1	
	>5 days	31	11	0.27(0.18-0.41) *	**0.56(0. 35-0.88)** **	**0.012**
Danger sign	Yes	58	18	1	1	
	No	202	27	2.14(1.58-2.89) *	1.48(1.07-2.05) **	**0.017**
Comorbidity	Yes	91	27	1	**1**	
	No	169	18	4.85(3.54-6.64) *	3.02(2.15-4.22) **	**0.000**
Oxygen therapy	Yes	153	22	1	1	
	No	107	23	2.38(1.83-3.10) *	1.71(1.20-2.41) **	**0.003**

**Note**: * P value<0.25, **P value<0.05, 1 = Reference.

## Discussion

This study was conducted to assess the time to recovery and its predictors among pediatric patients with severe community-acquired pneumonia admitted to public general hospitals of Tigray, Ethiopia. Predictors associated with the time to recovery from SCAP were rural residence, late presentation for seeking care, absence of danger signs, absence of comorbidity and not receiving oxygen therapy.

This study found that the overall incidence of recovery rate from SCAP was 16.30 per 100 person days observation (95% CI: 14.58, 18.01). This is lower than the study findings in Public Hospitals of Central and North Gondar Zones, Ethiopia [21] and Benishangul-Gumuz public hospitals [34], where incidence rate were 26.71 and 19.69 per 100 person-day observations respectively. This difference may be because the age groups in the studies are different. In this study, the age range includes children under 15 years old, while the previous studies focused on children under 5 years old. Additionally, the number of participants in this study was smaller than in those studies, and they used a retrospective design, while this study used a prospective design. Moreover, this finding showed that higher recovery rate than a study conducted in Asella Referral and Teaching Hospital, Oromia, Ethiopia [22], where the incidence rate was 13.089, per 100-person day observations. This difference might be due to variation in service, where referral hospital receives typically complicated patients to give advanced care, leading to lower recovery rate.

In this study, the median time to recovery of SCAP was 5 days (IQR:3–6). This finding is in line with the findings of South West Region governmental hospitals which were 5 days [36]. This may be due to similarities in age groups and study design. This is lower than the findings of the study in Adis Abeba in selected public hospitals where the median time to recovery was 6 days [23]. This difference may be due to the significant involvement of non-governmental organizations (NGOs) in the region because of the conflict. These NGOs provide free treatment for pediatric patients with SCAP. Their accessible care plays a crucial role in improving recovery times and health outcomes for children affected by SCAP.

Pediatric patients in rural areas had a 31% lower recovery rate from SCAP than those in urban areas (AHR = 0.69; 95% CI ((0.53–0.90). This finding is consistent with the findings of Public Hospitals of Central and North Gondar Zones and South West Region governmental hospitals, Ethiopia [21,37]. This might be because rural areas experience a higher rate of undernutrition compared to urban areas. Undernutrition weakens the immune system, which delays recovery [38].

Pediatric patients who were late presentation in seeking care had a 44% lower recovery rate from SCAP than early presenters (AHR = 0.56; 95% CI (0.35–0.88). This finding is consistent with studies conducted in Public Hospitals of Central and North Gondar Zones, Benishangul-Gumuz public hospitals, Debre Markos referral hospital, and South Wollo zone public hospitals which was 29%,33%,36 and 79%)respectively [1,21,24,34]. This may be due to delayed diagnosis and treatment of SCAP, which can lead to disease progression, secondary infections, and complications. These often result in longer hospital stays, more intensive treatment, poorer clinical outcomes, prolonged recovery, and increased risk of mortality, including complications such as respiratory failure and sepsis [39].

Pediatric patients without comorbidities had a 3.02 times faster recovery rate from SCAP than their counterparts (AHR = 3.02; 95% CI: 2.15–4.22). This finding in line with studies done in Debre Markos referral hospital, Public Hospitals of Central and North Gondar Zones and Asella Referral and Teaching Hospital, Ethiopia [1,21,22]. This might be due to the presence of comorbidities, which can delay recovery. These additional conditions place a significant burden on the body and weaken the immune system, reducing its ability to effectively fight severe community-acquired pneumonia (SCAP) and associated secondary infections. As a result, children with comorbidities are more likely to experience disease progression, complications, longer hospital stays, and delayed recovery [40]..

Pediatric patients without danger signs at admission had a 1.48 times higher recovery rate from SCAP compared to their counterparts (AHR = 1.48; 95% CI: 1.07–2.05). This finding is consistent with studies conducted at Debre Markos Referral Hospital, public hospitals of the Central and North Gondar Zones, and Asella Referral and Teaching Hospital, Ethiopia [1,21,22]. This may be due to differences in disease severity. As the disease progresses, children develop danger signs, and the time required for recovery usually increases. In severe cases of pneumonia, the body responds more complexly, leading to inflammation, respiratory distress, and potential organ dysfunction. This can result in prolonged symptoms, greater tissue damage, and delayed healing. Consequently, patients may require more intensive medical care, such as longer hospital stays or additional treatments, which can further delay recovery [41].

Pediatric patients who did not receive oxygen therapy had a 1.71 times higher recovery rate from SCAP compared to those who received oxygen therapy (AHR = 1.71; 95% CI: 1.20–2.41). This finding is consistent with a study conducted at Asella Referral and Teaching Hospital, Oromia Regional State, Ethiopia [22]. This may be because children who require oxygen therapy generally present with more severe illness and respiratory distress, indicating advanced disease. Oxygen therapy is an essential supportive treatment for stabilizing hypoxemic patients and enabling further management of SCAP. However, the need for oxygen often reflects greater disease severity and requires close monitoring, which may be associated with prolonged hospitalization. Consequently, this may delay recovery compared to patients who do not require oxygen therapy [42].

### Limitations of the study

Data Collection Challenges: Difficulties in collecting and recording data can introduce errors and short follow-up period.

## Conclusions

The median recovery time of patients with SCAP is considerably high as to previous studies conducted in Ethiopia. This may be due to post-conflict health service disruptions, shortages of essential medicines, limited diagnostic and treatment capacity, delayed care-seeking, higher comorbidity, and poor nutritional status. Rural residence, late presentation, absence of danger signs, absence of comorbidity, and not receiving oxygen therapy were significant predictors of time to recovery.

### Recommendations

**Regional Health Bureaus:** Develop a regional SCAP strategy that addresses research findings on delayed care among rural residents, late presentation, danger signs, comorbidities, and the need for oxygen therapy, considering post-conflict health system rebuilding and training of health professionals.

**Hospital Managers:** Ensure proper supervision of healthcare providers managing children with SCAP in the ward and work to reduce recovery time by addressing factors affecting time to recovery.

**Healthcare Providers:** When managing children with SCAP, prioritize factors known to influence recovery time and provide appropriate attention to high-risk cases.

**Community:** Parents and caregivers should seek timely medical attention for sick children at the nearest health facility.

**For policymakers:** Strengthen healthcare system capacity by training healthcare professionals, ensuring the continuous availability of essential medicines and oxygen, improving access to free, quality healthcare services, and strengthening child nutrition programs to improve recovery outcomes among children with SCAP.

## Supporting information

S1 File Data set. (XLSX)

## Abbreviations

AHR: Adjusted Hazard Ratio
CHR: Crude Hazard Ratios
CAP: Community-acquired pneumonia
IQR: Interquartile range
RR: Respiratory rate
SCAP: Severe community-acquired pneumonia
