# Supplementary material for: Time to recovery and its predictors among pediatric patients with severe community-acquired pneumonia admitted to public general hospitals in Tigray, Ethiopia, 2025: A prospective follow-up study
Source: PLoS One. 2026 Sep 24;21(9):e0358705. doi: 10.1371/journal.pone.0358705 (PMC13606997; doi:10.1371/journal.pone.0358705)
Supplement: Supplementary file 1 [file pone.0358705.s002.docx]

This manuscript addresses an important public health issue: recovery time and predictors of recovery among children with severe community-acquired pneumonia (SCAP) in Ethiopia. The topic is relevant, particularly in low-resource settings where prolonged hospitalization contributes to healthcare burden. The prospective follow-up design, use of survival analysis, and inclusion of multiple hospitals are strengths.

However, the following were observed:

**TOPIC**

- According to your topic “Time to recovery **and its predictors of severe community-acquired pneumonia** among pediatric patients admitted in public general hospitals of Tigray, Ethiopia 2024/2025”, are you stating that time to recovery is a predictor of severe community-acquired pneumonia? Kindly clarify this.

What I can deduce here is that your statement implies that the predictors are predictors **of pneumonia**, not predictors of recovery time.

- Under your Abstract result section, Line 39, there is a grammatical error: “**The recovered of pediatric patients were 85.2%**, with a median recovery time of 5 days”.

- Line 40 to 42 “Being rural residence **(AHR=0.69;95%CI(0.53-0.90),** late presenter to seeking care (AHR=0.56; 95% CI(0.35-0.88), danger sign **(AHR=1.48; 95% CI: 1.07–2.05)),** comorbidity **(AHR =3.02; 95% CI: 2.15–4.22)** and Oxygen **therapy (AHR = 1.71; 95% CI: 1.20-2.41),** were significant predictors”.

There is a **major issue of interpretation consistency** in this result. In time-to-recovery studies, predictors such as **comorbidity, oxygen therapy, danger signs, and late presentation** are usually expected to reduce the chance of recovery (AHR <1) because they indicate severe disease.

However, kindly clarify,

- Was the event coded as “recovery = 1” and “not recovered/censored = 0”?
- Did they accidentally interpret AHR as a risk factor for poor outcome instead of recovery?
- Were variables coded inversely (e.g., oxygen therapy coded as no oxygen = 1)?
- Was the proportional hazard assumption checked?
- It was stated in line 131 that the study was conducted in public general hospitals in Tigray, Ethiopia. “no prospective follow-up study conducted on time to recovery… in Tigray.”

However, the manuscript does not sufficiently explain: Why Tigray may differ from other Ethiopian regions. How recent conflict, healthcare disruption, or access issues may influence pneumonia outcomes. Why recovery time is a more meaningful outcome than mortality alone.

- Line 135 shows that the study was conducted from December 20, 2024, to February 20, 2025, the study period looks short for a prospective study.
- Line 145, under your inclusion criteria, it was noted that: all Children aged above 2 months to under 15 years with SCAP who were admitted on pediatric ward to the selected public general hospitals of Tigray during the study period were included, but, it was noted in your background that the highest rate of pneumonia is in children under five, why were children over 5 years up to 15 years included? In fact, we can say that ages 10 and above are already adolescents.
- Under sample size determination, lines 158 to 163, it was stated that: Finally, the **sample size was 183** …… thus, by adding a non-response rate of 10% and 1.5 design effect, **the final sample size for this study was 305.** Two sample sizes were mentioned. How did we come about these figures? What was the initial calculated sample? How was the final sample derived? Why was a design effect used in a hospital-based study? Was the study powered for Cox regression predictors or only overall recovery? Kindly show the calculation.
- Lines 177 to 179 state that: after allocation, finally, a systematic random sampling method (K = N/n = 422/305 ≈ 1.4 ≈ 1) was used. The first participant was selected randomly, and then every Kth interval (every 1st interval) was chosen as a study participant during admission.

This means essentially every eligible child was selected. Calling this “systematic random sampling” is questionable. If K≈1, this is effectively **consecutive sampling**, not systematic random sampling.

- Lines 191 to 192, under operational definition of terms, The definition of recovery is “Children improved from SCAP as declared by the clinician/physician.” This is a major methodological limitation.

Recovery depends on the clinician's judgement. No objective criteria were provided.

- The final model excludes nutritional status despite strong associations in Kaplan-Meier analysis.

For example, underweight, stunting, and wasting showed markedly longer recovery times.

However, they disappeared in the adjusted model.

The authors should explain whether they were confounded, whether collinearity existed, and whether they were removed intentionally.

- Tables: In Table 5, Several variables show strong associations in the Kaplan-Meier analysis but become non-significant in the Cox regression. The manuscript should explain this transition. **Table 6:** The table is difficult to interpret because reference groups are not clearly explained, and hazard ratio interpretation is confusing. Compare your value of comorbidities in your abstract with the one in Table 6, check others as well, and confidence intervals should be consistently formatted.
- Limited discussion of limitations. The limitation section only states: “Data collection challenges…” Important limitations include: Single region setting. Hospital-based design. Possible residual confounding. Short follow-up period. Potential measurement bias.
