# Supplementary material for: Time to recovery and its predictors among pediatric patients with severe community-acquired pneumonia admitted to public general hospitals in Tigray, Ethiopia, 2025: A prospective follow-up study
Source: PLoS One. 2026 Sep 24;21(9):e0358705. doi: 10.1371/journal.pone.0358705 (PMC13606997; doi:10.1371/journal.pone.0358705)
Supplement: Supplementary file 3 [file pone.0358705.s005.docx]

**Response to Reviewers**

**PONE-D-25-63360**

**Title: Time to recovery and its predictors among pediatric patients with severe community-acquired pneumonia admitted to public general hospitals of Tigray, Ethiopia, 2025: a prospective follow -up study**

**PLOS ONE**

**Point by point response to Reviewers Comments: -**

| **Sr. No** | **Reviewers' Comments** | **Response to Comments** |
| --- | --- | --- |
|  | **Academic Editor comments** |  |
| 1 | Please ensure that your manuscript meets PLOS ONE's style requirements, including those for file naming. The PLOS ONE style templates can be found at <https://journals.plos.org/plosone/s/file?id=wjVg/PLOSOne_formatting_sample_main_body.pdf> and [https://journals.plos.org/plosone/s/file?id=ba62/PLOSOne_formatting_sample_title_authors_affiliations.pdf](https://track.editorialmanager.com/CL0/https:%2F%2Fjournals.plos.org%2Fplosone%2Fs%2Ffile%3Fid=ba62%2FPLOSOne_formatting_sample_title_authors_affiliations.pdf/1/010f019ef7c3ee2a-f112061e-8f9e-4afa-9a5c-e2a1f46b434c-000000/IEgAonC5k7TJc43nFYitnji6BrKwLteg7cbzaWLFlsE=258) | We accept the comment and we made corrections according the PLOS ONE guideline. |
| 2 | Please provide a complete Data Availability Statement in the submission form, ensuring you include all necessary access information or a reason for why you are unable to make your data freely accessible. If your research concerns only data provided within your submission, please write "All data are in the manuscript and/or supporting information files" as your Data Availability Statement. | The comment was well taken, and we ensured that we provided the necessary data and information according to your requirements. |
| 3 | Please include captions for your Supporting Information files at the end of your manuscript, and update any in-text citations to match accordingly. Please see our Supporting Information guidelines for more information: http://journals.plos.org/plosone/s/supporting-information. | we accepted the comment and provided the requested data set as required. |
| 4 | If the reviewer comments include a recommendation to cite specific previously published works, please review and evaluate these publications to determine whether they are relevant and should be cited. There is no requirement to cite these works unless the editor has indicated otherwise. | The comment was well taken, and we uploaded the required data as Supporting Information files. |
|  | **Reviewer#1** |  |
|  | **General comments: on topic** |  |
| 1 | The phrase “pediatric patient” should be plural (“pediatric patients”), and “community acquired” should be hyphenated as “community-acquired.” The title lacks information on the study design and setting, which are important for academic clarity (e.g., prospective follow-up study, hospital-based, geographic location, “during 2024–2025”). The title can be more concise and impactful by removing unnecessary words and restructuring for smoother flow. | We accept the comment. We made correction accordingly. |
|  | **Abstract:** |  |
| 2 | The abstract is well structured, but minor grammatical issues and inconsistent phrasing (e.g., “The recovered of pediatric patients,” repeated use of “pediatric patient”) should be corrected to improve clarity and readability. The results section would benefit from clearer interpretation of hazard ratios, particularly where higher AHRs indicate faster recovery (e.g., danger signs and comorbidity), to avoid potential confusion for readers. | We accept the comment. We made correction accordingly |
|  | **Introduction:** |  |
| 3 | It is comprehensive but overly lengthy and repetitive in places, particularly in the global and national burden sections. Condensing overlapping statistics and streamlining similar arguments would improve flow and maintain reader engagement. There are frequent grammatical errors, inconsistent punctuation, and formatting issues (e.g., spacing, line breaks, symbols, and citation placement) that reduce clarity and professionalism. Definitions and clinical criteria could be simplified and presented more consistently. | We accept the comment. We made correction accordingly |
|  | **Methods:** |  |
| 4 | The sample size section presents conflicting figures (183 vs. 305) and repeats explanations, making it unclear how the final sample size was definitively derived and justified. Recovery from SCAP is based on clinician declaration, but no standardized clinical criteria or inter-observer reliability measures are described, which may introduce measurement bias. | We accept the comment. We made correction accordingly |
|  | **Result** |  |
| 5 | The Results section provides detailed and well-organized descriptive, survival, and regression analyses, offering a strong quantitative overview of SCAP recovery patterns. However, results are overly descriptive with limited concise interpretation, and key multivariable findings are not clearly or consistently summarized. | We accept the comment. We made correction accordingly |
|  | **Discussion:** |  |
| 6 | The explanations for differences with other studies are mostly general and not supported by specific data from this study. The discussion repeats the results a lot instead of deeply analyzing why these factors affect recovery or what the findings mean for practice and public health. | We accept the comment. We made correction accordingly |
|  | **Conclusions and recommendations:** |  |
| 7 | The conclusions mainly repeat the study findings and do not clearly explain why the recovery time is higher than in previous studies, which limits deeper understanding of the results. In addition, the recommendations are very general and lack specific, practical actions linked to the study findings, making it difficult for policymakers, health managers, and providers to apply them effectively in real settings. | We accept the comment. We made correction accordingly |
|  | **Reviewer #2:** |  |
|  | **Topic** |  |
| 1 | According to your topic “Time to recovery and its predictors of severe community-acquired pneumonia among pediatric patients admitted in public general hospitals of Tigray, Ethiopia 2024/2025”, are you stating that time to recovery is a predictor of severe community-acquired pneumonia? Kindly clarify this. What I can deduce here is that your statement implies that the predictors are predictors of pneumonia, not predictors of recovery time. | We accept the comment. We made correction accordingly |
|  | **Abstracts** |  |
| 2 | Under your Abstract result section, Line 39, there is a grammatical error: “The recovered of pediatric patients were 85.2%, with a median recovery time of 5 days”.  Line 40 to 42 “Being rural residence (AHR=0.69;95%CI(0.53-0.90), late presenter to seeking care (AHR=0.56; 95% CI(0.35-0.88), danger sign (AHR=1.48; 95% CI: 1.07–2.05)), comorbidity (AHR =3.02; 95% CI: 2.15–4.22) and Oxygen therapy (AHR = 1.71; 95% CI: 1.20-2.41), were significant predictors”. There is a major issue of interpretation consistency in this result. In time-to-recovery studies, predictors such as comorbidity, oxygen therapy, danger signs, and late presentation are usually expected to reduce the chance of recovery (AHR <1) because they indicate severe disease. | We accept the comment. We made correction accordingly |
|  | However, kindly clarify, Was the event coded as “recovery = 1” and “not recovered/censored = 0”? | Yes: recovery = 1 and censored = 0 |
|  | Did they accidentally interpret AHR as a risk factor for poor outcome instead of recovery? | We do not interpret the adjusted hazard ratio (AHR) as a risk factor for poor outcome; rather, we use it to assess recovery. |
|  | Were variables coded inversely (e.g., oxygen therapy coded as no oxygen = 1)? | Oxygen therapy coded as yes = 1 used as reference |
|  | Was the proportional hazard assumption checked? | Yes |
|  | Methods |  |
|  | It was stated in line 131 that the study was conducted in public general hospitals in Tigray, Ethiopia. “No prospective follow-up study conducted on time to recovery… in Tigray.” However, the manuscript does not sufficiently explain: Why Tigray may differ from other Ethiopian regions. How recent conflict, healthcare disruption, or access issues may influence pneumonia outcomes. Why recovery time is a more meaningful outcome than mortality alone. | We accept the comment. We made correction accordingly |
|  | Line 135 shows that the study was conducted from December 20, 2024, to February 20, 2025, the study period looks short for a prospective study. | Yes, because the target sample size has already been achieved. |
|  | • Line 145, under your inclusion criteria, it was noted that: all Children aged above 2 months to under 15 years with SCAP who were admitted on pediatric ward to the selected public general hospitals of Tigray during the study period were included, but, it was noted in your background that the highest rate of pneumonia is in children under five, why were children over 5 years up to 15 years included? In fact, we can say that ages 10 and above are already adolescents. | In many studies, children under five years of age are more commonly affected, while those older than five years are rarely included in most studies on SCAP. |
|  | Under sample size determination, lines 158 to 163, it was stated that: Finally, the sample size was 183 …… thus, by adding a non-response rate of 10% and 1.5 design effect, the final sample size for this study was 305. Two sample sizes were mentioned. How did we come about these figures? What was the initial calculated sample? How was the final sample derived? | We accept the comment. We made correction accordingly |
|  | Why was a design effect used in a hospital-based study? Was the study powered for Cox regression predictors or only overall recovery? Kindly show the calculation. | The sampling procedure started from regional to zonal and then to general hospitals and also in order to obtain a larger sample size and ensure adequate statistical power. |
|  | Lines 177 to 179 state that: after allocation, finally, a systematic random sampling method (K = N/n = 422/305 ≈ 1.4 ≈ 1) was used. The first participant was selected randomly, and then every Kth interval (every 1st interval) was chosen as a study participant during admission. This means essentially every eligible child was selected. Calling this “systematic random sampling” is questionable. If K≈1, this is effectively consecutive sampling, not systematic random sampling. | We accept the comment and have made the correction accordingly. We used every 2^nd^ interval; the previous wording was a writing error, which has now been corrected. |
|  | Lines 191 to 192, under operational definition of terms, the definition of recovery is “Children improved from SCAP as declared by the clinician/physician.” This is a major methodological limitation. Recovery depends on the clinician's judgement. No objective criteria were provided. | Recovery was determined by the treating physician based on standard clinical discharge criteria documented in the medical record. |
|  | **Results** |  |
| 3 | The final model excludes nutritional status despite strong associations in Kaplan-Meier analysis. For example, underweight, stunting, and wasting showed markedly longer recovery times. However, they disappeared in the adjusted model. The authors should explain whether they were confounded, whether collinearity existed, and whether they were removed intentionally. | We accept the comment. However, we only reported the results of the multivariable analysis, and the three variables were included in the final model but were not significantly associated with the outcome variable. |
|  | Tables: In Table 5, Several variables show strong associations in the Kaplan-Meier analysis but become non-significant in the Cox regression. The manuscript should explain this transition. | We accept the comment. We made correction accordingly |
|  | Table 6: The table is difficult to interpret because reference groups are not clearly explained, and hazard ratio interpretation is confusing. Compare your value of comorbidities in your abstract with the one in Table 6, check others as well, and confidence intervals should be consistently formatted. | We accept the comment. We made correction accordingly |
| 4 | **Discussion of limitations.** |  |
|  | The limitation section only states: “Data collection challenges…” Important limitations include: Single region setting. Hospital-based design. Possible residual confounding. Short follow-up period. Potential measurement bias | We accept the comment. We made correction accordingly |

We would be glad to respond to any further questions and comments that you may have.
