# Supplementary material for: Time to recovery and its predictors among pediatric patients with severe community-acquired pneumonia admitted to public general hospitals in Tigray, Ethiopia, 2025: A prospective follow-up study
Source: PLoS One. 2026 Sep 24;21(9):e0358705. doi: 10.1371/journal.pone.0358705 (PMC13606997; doi:10.1371/journal.pone.0358705)
Supplement: Supplementary file 4 [file pone.0358705.s006.docx]

**Response to Reviewers**

**PONE-D-25-63360**

**Title: Time to recovery and its predictors among pediatric patients with severe community-acquired pneumonia admitted to public general hospitals of Tigray, Ethiopia, 2025: a prospective follow -up study**

**PLOS ONE**

**Point by point response to Reviewers Comments: -**

| **Sr. No** | **Reviewers' Comments** | **Response to Comments** |
| --- | --- | --- |
|  | **Methods:** |  |
| 4 | Review Comments to the Author  Please use the space provided to explain your answers to the questions above. You may also include additional comments for the author, including concerns about dual publication, research ethics, or publication ethics. (Please upload your review as an attachment if it exceeds 20,000 characters)  Reviewer #2: Thank you for revising the manuscript and addressing most of my previous comments. I appreciate the authors' efforts to improve the manuscript. However, one methodological issue remains unresolved and requires clarification before the manuscript can be considered for publication. Comment on the Sampling Procedure The explanation provided regarding the sampling procedure remains mathematically inconsistent. The manuscript indicates that the total sampling frame was N = 424 and the required sample size was n = 305, giving a sampling interval of: K = N/n = 422/305 ≈ 1.38. The authors now state that they used every 2nd interval, but this is not supported by the reported figures. Selecting every second eligible participant from a population of 422 would yield approximately 211 participants, not the reported sample size of 305. Therefore, the current description of the sampling method remains unclear. The authors should either: 1. Provide the correct sampling frame and calculations if systematic random sampling was indeed employed; or 2. Revise the methodology to accurately reflect the actual recruitment procedure. If nearly every eligible child admitted during the study period was enrolled until the required sample size was reached, the appropriate description would be consecutive recruitment (or consecutive sampling) rather than systematic random sampling. Please ensure that the sampling method described is mathematically consistent with the reported sample size and sampling frame. | We accept the comment. We made correction accordingly. |

We would be glad to respond to any further questions and comments that you may have.
