# Supplementary material for: Time to recovery and its predictors among pediatric patients with severe community-acquired pneumonia admitted to public general hospitals in Tigray, Ethiopia, 2025: A prospective follow-up study
Source: PLoS One. 2026 Sep 24;21(9):e0358705. doi: 10.1371/journal.pone.0358705 (PMC13606997; doi:10.1371/journal.pone.0358705)
Supplement: Peer review history

# Peer review: Decision Letter 0

**DOI:** 10.1371/journal.pone.0358705.r001

**Authors:** Kahsu Gebrekidan

23 Jun 2026

Dear Dr. Tekle,

Thank you for submitting your manuscript to PLOS ONE. After careful consideration, we feel that it has merit but does not fully meet PLOS ONE’s publication criteria as it currently stands. Therefore, we invite you to submit a revised version of the manuscript that addresses the points raised during the review process.

Please submit your revised manuscript by  Aug 07 2026 11:59PM. If you will need more time than this to complete your revisions, please reply to this message or contact the journal office at plosone@plos.org. When you're ready to submit your revision, log on to https://www.editorialmanager.com/pone/ and select the 'Submissions Needing Revision' folder to locate your manuscript file.

- A letter that responds to each point raised by the academic editor and reviewer(s). You should upload this letter as a separate file labeled 'Response to Reviewers'.
- A marked-up copy of your manuscript that highlights changes made to the original version. You should upload this as a separate file labeled 'Revised Manuscript with Track Changes'.
- An unmarked version of your revised paper without tracked changes. You should upload this as a separate file labeled 'Manuscript'.

If you would like to make changes to your financial disclosure, please include your updated statement in your cover letter. Guidelines for resubmitting your figure files are available below the reviewer comments at the end of this letter.

If applicable, we recommend that you deposit your laboratory protocols in protocols.io to enhance the reproducibility of your results. Protocols.io assigns your protocol its own identifier (DOI) so that it can be cited independently in the future. For instructions see: https://journals.plos.org/plosone/s/submission-guidelines#loc-laboratory-protocols. Additionally, PLOS ONE offers an option for publishing peer-reviewed Lab Protocol articles, which describe protocols hosted on protocols.io. Read more information on sharing protocols at https://plos.org/protocols?utm_medium=editorial-email&utm_source=authorletters&utm_campaign=protocols.

As the corresponding author, your ORCID iD is verified in the submission system and will appear in the published article. PLOS supports the use of ORCID, and we encourage all coauthors to register for an ORCID iD and use it as well. Please encourage your coauthors to verify their ORCID iD within the submission system before final acceptance, as unverified ORCID iDs will not appear in the published article. *Only*  the individual author can complete the verification step; PLOS staff *cannot*  verify ORCID iDs on behalf of authors.

We look forward to receiving your revised manuscript.

Kind regards,

Kahsu Gebrekidan, Ph.D.

Academic Editor

PLOS One

Journal Requirements:

When submitting your revision, we need you to address these additional requirements.

1. Please ensure that your manuscript meets PLOS ONE's style requirements, including those for file naming. The PLOS ONE style templates can be found at

https://journals.plos.org/plosone/s/file?id=wjVg/PLOSOne_formatting_sample_main_body.pdf and

https://journals.plos.org/plosone/s/file?id=ba62/PLOSOne_formatting_sample_title_authors_affiliations.pdf

2. Please provide a complete Data Availability Statement in the submission form, ensuring you include all necessary access information or a reason for why you are unable to make your data freely accessible. If your research concerns only data provided within your submission, please write "All data are in the manuscript and/or supporting information files" as your Data Availability Statement.

3. Please include captions for your Supporting Information files at the end of your manuscript, and update any in-text citations to match accordingly. Please see our Supporting Information guidelines for more information: http://journals.plos.org/plosone/s/supporting-information.

4. If the reviewer comments include a recommendation to cite specific previously published works, please review and evaluate these publications to determine whether they are relevant and should be cited. There is no requirement to cite these works unless the editor has indicated otherwise.

[Note: HTML markup is below. Please do not edit.]

Reviewers' comments:

Reviewer's Responses to Questions

**Comments to the Author**

1. Is the manuscript technically sound, and do the data support the conclusions?

Reviewer #1: Partly

Reviewer #2: Partly

**********

2. Has the statistical analysis been performed appropriately and rigorously?

Reviewer #1: Yes

Reviewer #2: Yes

**********

3. Have the authors made all data underlying the findings in their manuscript fully available?

The PLOS Data policy

Reviewer #1: Yes

Reviewer #2: Yes

**********

4. Is the manuscript presented in an intelligible fashion and written in standard English?

Reviewer #1: Yes

Reviewer #2: Yes

**********

Reviewer #1: Comments on Title:

The phrase “pediatric patient” should be plural (“pediatric patients”), and “community acquired” should be hyphenated as “community-acquired.” The title lacks information on the study design and setting, which are important for academic clarity (e.g., prospective follow-up study, hospital-based, geographic location, “during 2024–2025”). The title can be more concise and impactful by removing unnecessary words and restructuring for smoother flow.

Comments on Abstract:

The abstract is well structured, but minor grammatical issues and inconsistent phrasing (e.g., “The recovered of pediatric patients,” repeated use of “pediatric patient”) should be corrected to improve clarity and readability. The results section would benefit from clearer interpretation of hazard ratios, particularly where higher AHRs indicate faster recovery (e.g., danger signs and comorbidity), to avoid potential confusion for readers.

Comments on introduction:

It is comprehensive but overly lengthy and repetitive in places, particularly in the global and national burden sections. Condensing overlapping statistics and streamlining similar arguments would improve flow and maintain reader engagement. There are frequent grammatical errors, inconsistent punctuation, and formatting issues (e.g., spacing, line breaks, symbols, and citation placement) that reduce clarity and professionalism. Definitions and clinical criteria could be simplified and presented more consistently.

Comments on Method:

The sample size section presents conflicting figures (183 vs. 305) and repeats explanations, making it unclear how the final sample size was definitively derived and justified. Recovery from SCAP is based on clinician declaration, but no standardized clinical criteria or inter-observer reliability measures are described, which may introduce measurement bias.

Comments on Result:

The Results section provides detailed and well-organized descriptive, survival, and regression analyses, offering a strong quantitative overview of SCAP recovery patterns. However results are overly descriptive with limited concise interpretation, and key multivariable findings are not clearly or consistently summarized.

Comments on discussions:

The explanations for differences with other studies are mostly general and not supported by specific data from this study. The discussion repeats the results a lot instead of deeply analyzing why these factors affect recovery or what the findings mean for practice and public health.

Comments on Conclusions and recommendations:

The conclusions mainly repeat the study findings and do not clearly explain why the recovery time is higher than in previous studies, which limits deeper understanding of the results. In addition, the recommendations are very general and lack specific, practical actions linked to the study findings, making it difficult for policymakers, health managers, and providers to apply them effectively in real settings.

Reviewer #2: This manuscript addresses an important public health issue: recovery time and predictors of recovery among children with severe community-acquired pneumonia (SCAP) in Ethiopia. The topic is relevant, particularly in low-resource settings where prolonged hospitalization contributes to healthcare burden. The prospective follow-up design, use of survival analysis, and inclusion of multiple hospitals are strengths.

However, the following were observed in the manuscript:

TOPIC

• According to your topic “Time to recovery and its predictors of severe community-acquired pneumonia among pediatric patients admitted in public general hospitals of Tigray, Ethiopia 2024/2025”, are you stating that time to recovery is a predictor of severe community-acquired pneumonia? Kindly clarify this.

What I can deduce here is that your statement implies that the predictors are predictors of pneumonia, not predictors of recovery time.

• Under your Abstract result section, Line 39, there is a grammatical error: “The recovered of pediatric patients were 85.2%, with a median recovery time of 5 days”.

• Line 40 to 42 “Being rural residence (AHR=0.69;95%CI(0.53-0.90), late presenter to seeking care (AHR=0.56; 95% CI(0.35-0.88), danger sign (AHR=1.48; 95% CI: 1.07–2.05)), comorbidity (AHR =3.02; 95% CI: 2.15–4.22) and Oxygen therapy (AHR = 1.71; 95% CI: 1.20-2.41), were significant predictors”.

There is a major issue of interpretation consistency in this result. In time-to-recovery studies, predictors such as comorbidity, oxygen therapy, danger signs, and late presentation are usually expected to reduce the chance of recovery (AHR <1) because they indicate severe disease.

However, kindly clarify,

� Was the event coded as “recovery = 1” and “not recovered/censored = 0”?

� Did they accidentally interpret AHR as a risk factor for poor outcome instead of recovery?

� Were variables coded inversely (e.g., oxygen therapy coded as no oxygen = 1)?

� Was the proportional hazard assumption checked?

• It was stated in line 131 that the study was conducted in public general hospitals in Tigray, Ethiopia. “no prospective follow-up study conducted on time to recovery… in Tigray.”

However, the manuscript does not sufficiently explain: Why Tigray may differ from other Ethiopian regions. How recent conflict, healthcare disruption, or access issues may influence pneumonia outcomes. Why recovery time is a more meaningful outcome than mortality alone.

• Line 135 shows that the study was conducted from December 20, 2024, to February 20, 2025, the study period looks short for a prospective study.

• Line 145, under your inclusion criteria, it was noted that: all Children aged above 2 months to under 15 years with SCAP who were admitted on pediatric ward to the selected public general hospitals of Tigray during the study period were included, but, it was noted in your background that the highest rate of pneumonia is in children under five, why were children over 5 years up to 15 years included? In fact, we can say that ages 10 and above are already adolescents.

• Under sample size determination, lines 158 to 163, it was stated that: Finally, the sample size was 183 …… thus, by adding a non-response rate of 10% and 1.5 design effect, the final sample size for this study was 305. Two sample sizes were mentioned. How did we come about these figures? What was the initial calculated sample? How was the final sample derived? Why was a design effect used in a hospital-based study? Was the study powered for Cox regression predictors or only overall recovery? Kindly show the calculation.

• Lines 177 to 179 state that: after allocation, finally, a systematic random sampling method (K = N/n = 422/305 ≈ 1.4 ≈ 1) was used. The first participant was selected randomly, and then every Kth interval (every 1st interval) was chosen as a study participant during admission.

This means essentially every eligible child was selected. Calling this “systematic random sampling” is questionable. If K≈1, this is effectively consecutive sampling, not systematic random sampling.

• Lines 191 to 192, under operational definition of terms, The definition of recovery is “Children improved from SCAP as declared by the clinician/physician.” This is a major methodological limitation.

Recovery depends on the clinician's judgement. No objective criteria were provided.

• The final model excludes nutritional status despite strong associations in Kaplan-Meier analysis.

For example, underweight, stunting, and wasting showed markedly longer recovery times.

However, they disappeared in the adjusted model.

The authors should explain whether they were confounded, whether collinearity existed, and whether they were removed intentionally.

• Tables: In Table 5, Several variables show strong associations in the Kaplan-Meier analysis but become non-significant in the Cox regression. The manuscript should explain this transition. Table 6: The table is difficult to interpret because reference groups are not clearly explained, and hazard ratio interpretation is confusing. Compare your value of comorbidities in your abstract with the one in Table 6, check others as well, and confidence intervals should be consistently formatted.

• Limited discussion of limitations. The limitation section only states: “Data collection challenges…” Important limitations include: Single region setting. Hospital-based design. Possible residual confounding. Short follow-up period. Potential measurement bias.

**********

what does this mean?). If published, this will include your full peer review and any attached files.

If you choose “no”, your identity will remain anonymous but your review may still be made public.

**Do you want your identity to be public for this peer review?**  For information about this choice, including consent withdrawal, please see our Privacy Policy

Reviewer #1: **Yes:** Abu Sayeed Md. Abdullah,

PhD Candidate, Mid Sweden University (MIUN), Sweden; and

Deputy Director, RCH, CIPRB, Bangladesh

Reviewer #2: No

**********

[NOTE: If reviewer comments were submitted as an attachment file, they will be attached to this email and accessible via the submission site. Please log into your account, locate the manuscript record, and check for the action link "View Attachments". If this link does not appear, there are no attachment files.]

To ensure your figures meet our technical requirements, please review our figure guidelines: https://journals.plos.org/plosone/s/figures

You may also use PLOS’s free figure tool, NAAS, to help you prepare publication quality figures: https://journals.plos.org/plosone/s/figures#loc-tools-for-figure-preparation.

NAAS will assess whether your figures meet our technical requirements by comparing each figure against our figure specifications.

Attachment Submitted filename: Review SCAP.docx

# Author comment: Author response to Decision Letter 1

**DOI:** 10.1371/journal.pone.0358705.r002

16 Jul 2026

1.Please ensure that your manuscript meets PLOS ONE's style requirements, including those for file naming. The PLOS ONE style templates can be found at

https://journals.plos.org/plosone/s/file?id=wjVg/PLOSOne_formatting_sample_main_body.pdf and

https://journals.plos.org/plosone/s/file?id=ba62/PLOSOne_formatting_sample_title_authors_affiliations.pdf

answer: We accept the comment and we made corrections according to the PLOS ONE guideline.

2. Please provide a complete Data Availability Statement in the submission form, ensuring you include all necessary access information or a reason for why you are unable to make your data freely accessible. If your research concerns only data provided within your submission, please write "All data are in the manuscript and/or supporting information files" as your Data Availability Statement.

Answer: All data are included in the manuscript and supporting information files.

3.Please include captions for your Supporting Information files at the end of your manuscript, and update any in-text citations to match accordingly. Please see our Supporting Information guidelines for more information: http://journals.plos.org/plosone/s/supporting-information.

Answer: we accepted the comment and provided the requested data set as required.

4.If the reviewer comments include a recommendation to cite specific previously published works, please review and evaluate these publications to determine whether they are relevant and should be cited. There is no requirement to cite these works unless the editor has indicated otherwise.

Answer: The comment was well taken, and we uploaded the required data as Supporting Information files.

Reviewer#1

1.The phrase “pediatric patient” should be plural (“pediatric patients”), and “community acquired” should be hyphenated as “community-acquired.” The title lacks information on the study design and setting, which are important for academic clarity (e.g., prospective follow-up study, hospital-based, geographic location, “during 2024–2025”). The title can be more concise and impactful by removing unnecessary words and restructuring for smoother flow.

Answer: We accept the comment. We made correction accordingly.

2.The abstract is well structured, but minor grammatical issues and inconsistent phrasing (e.g., “The recovered of pediatric patients,” repeated use of “pediatric patient”) should be corrected to improve clarity and readability. The results section would benefit from clearer interpretation of hazard ratios, particularly where higher AHRs indicate faster recovery (e.g., danger signs and comorbidity), to avoid potential confusion for readers.

Answer: We accept the comment. We made correction accordingly

3.It is comprehensive but overly lengthy and repetitive in places, particularly in the global and national burden sections. Condensing overlapping statistics and streamlining similar arguments would improve flow and maintain reader engagement. There are frequent grammatical errors, inconsistent punctuation, and formatting issues (e.g., spacing, line breaks, symbols, and citation placement) that reduce clarity and professionalism. Definitions and clinical criteria could be simplified and presented more consistently.

Answer: We accept the comment. We made correction accordingly

4.The sample size section presents conflicting figures (183 vs. 305) and repeats explanations, making it unclear how the final sample size was definitively derived and justified. Recovery from SCAP is based on clinician declaration, but no standardized clinical criteria or inter-observer reliability measures are described, which may introduce measurement bias.

Answer: We accept the comment. We made correction accordingly

5.The Results section provides detailed and well-organized descriptive, survival, and regression analyses, offering a strong quantitative overview of SCAP recovery patterns. However, results are overly descriptive with limited concise interpretation, and key multivariable findings are not clearly or consistently summarized.

Answer: We accept the comment. We made correction accordingly

6.The explanations for differences with other studies are mostly general and not supported by specific data from this study. The discussion repeats the results a lot instead of deeply analyzing why these factors affect recovery or what the findings mean for practice and public health.

Answer: We accept the comment. We made correction accordingly

7.The conclusions mainly repeat the study findings and do not clearly explain why the recovery time is higher than in previous studies, which limits deeper understanding of the results. In addition, the recommendations are very general and lack specific, practical actions linked to the study findings, making it difficult for policymakers, health managers, and providers to apply them effectively in real settings.

Answer: We accept the comment. We made correction accordingly

Reviewer #2:

1.According to your topic “Time to recovery and its predictors of severe community-acquired pneumonia among pediatric patients admitted in public general hospitals of Tigray, Ethiopia 2024/2025”, are you stating that time to recovery is a predictor of severe community-acquired pneumonia? Kindly clarify this.

What I can deduce here is that your statement implies that the predictors are predictors of pneumonia, not predictors of recovery time.

Answer: We accept the comment. We made correction accordingly

2.Under your Abstract result section, Line 39, there is a grammatical error: “The recovered of pediatric patients were 85.2%, with a median recovery time of 5 days”.

Line 40 to 42 “Being rural residence (AHR=0.69;95%CI(0.53-0.90), late presenter to seeking care (AHR=0.56; 95% CI(0.35-0.88), danger sign (AHR=1.48; 95% CI: 1.07–2.05)), comorbidity (AHR =3.02; 95% CI: 2.15–4.22) and Oxygen therapy (AHR = 1.71; 95% CI: 1.20-2.41), were significant predictors”.

There is a major issue of interpretation consistency in this result. In time-to-recovery studies, predictors such as comorbidity, oxygen therapy, danger signs, and late presentation are usually expected to reduce the chance of recovery (AHR <1) because they indicate severe disease.

Answer: We accept the comment. We made correction accordingly

3.However, kindly clarify, Was the event coded as “recovery = 1” and “not recovered/censored = 0”?

Answer: Yes: recovery = 1 and censored = 0

4.Did they accidentally interpret AHR as a risk factor for poor outcome instead of recovery?

Answer: We do not interpret the adjusted hazard ratio (AHR) as a risk factor for poor outcome; rather, we use it to assess recovery.

5.Were variables coded inversely (e.g., oxygen therapy coded as no oxygen = 1)?

Answer: Oxygen therapy coded as yes = 1 used as reference

6.Was the proportional hazard assumption checked?

Answer: Yes

7.It was stated in line 131 that the study was conducted in public general hospitals in Tigray, Ethiopia. “No prospective follow-up study conducted on time to recovery… in Tigray.”

However, the manuscript does not sufficiently explain: Why Tigray may differ from other Ethiopian regions. How recent conflict, healthcare disruption, or access issues may influence pneumonia outcomes. Why recovery time is a more meaningful outcome than mortality alone.

Answer :We accept the comment. We made correction accordingly

8.Line 135 shows that the study was conducted from December 20, 2024, to February 20, 2025, the study period looks short for a prospective study.

Answer: Yes, because the target sample size has already been achieved

9.Line 145, under your inclusion criteria, it was noted that: all Children aged above 2 months to under 15 years with SCAP who were admitted on pediatric ward to the selected public general hospitals of Tigray during the study period were included, but, it was noted in your background that the highest rate of pneumonia is in children under five, why were children over 5 years up to 15 years included? In fact, we can say that ages 10 and above are already adolescents.

Answer: In many studies, children under five years of age are more commonly affected, while those older than five years are rarely included in most studies on SCAP.

10.Under sample size determination, lines 158 to 163, it was stated that: Finally, the sample size was 183 …… thus, by adding a non-response rate of 10% and 1.5 design effect, the final sample size for this study was 305. Two sample sizes were mentioned. How did we come about these figures? What was the initial calculated sample? How was the final sample derived?

Answer: We accept the comment. We made correction accordingly

11.Why was a design effect used in a hospital-based study? Was the study powered for Cox regression predictors or only overall recovery? Kindly show the calculation.

Answer: The sampling procedure started from regional to zonal and then to general hospitals and also in order to obtain a larger sample size and ensure adequate statistical power.

12.Lines 177 to 179 state that: after allocation, finally, a systematic random sampling method (K = N/n = 422/305 ≈ 1.4 ≈ 1) was used. The first participant was selected randomly, and then every Kth interval (every 1st interval) was chosen as a study participant during admission.

This means essentially every eligible child was selected. Calling this “systematic random sampling” is questionable. If K≈1, this is effectively consecutive sampling, not systematic random sampling

Answer: We accept the comment and have made the correction accordingly. We used every 2nd interval; the previous wording was a writing error, which has now been corrected.

13.Lines 191 to 192, under operational definition of terms, the definition of recovery is “Children improved from SCAP as declared by the clinician/physician.” This is a major methodological limitation. Recovery depends on the clinician's judgement. No objective criteria were provided.

Answer: Recovery was determined by the treating physician based on standard clinical discharge criteria documented in the medical record.

14.The final model excludes nutritional status despite strong associations in Kaplan-Meier analysis.

For example, underweight, stunting, and wasting showed markedly longer recovery times.

However, they disappeared in the adjusted model.

The authors should explain whether they were confounded, whether collinearity existed, and whether they were removed intentionally.

Answer: We accept the comment. However, we only reported the results of the multivariable analysis, and the three variables were included in the final model but were not significantly associated with the outcome variable.

15.Tables: In Table 5, Several variables show strong associations in the Kaplan-Meier analysis but become non-significant in the Cox regression. The manuscript should explain this transition.

Answer: We accept the comment. We made correction accordingly

16.Table 6: The table is difficult to interpret because reference groups are not clearly explained, and hazard ratio interpretation is confusing. Compare your value of comorbidities in your abstract with the one in Table 6, check others as well, and confidence intervals should be consistently formatted.

Answer: We accept the comment. We made correction accordingly

17.The limitation section only states: “Data collection challenges…” Important limitations include: Single region setting. Hospital-based design. Possible residual confounding. Short follow-up period. Potential measurement bias

Answer: We accept the comment. We made correction accordingly

Attachment Submitted filename: Response to Reviewers.docx

# Peer review: Decision Letter 1

**DOI:** 10.1371/journal.pone.0358705.r003

**Authors:** Kahsu Gebrekidan

18 Aug 2026

Dear Dr. Tekle,

Please submit your revised manuscript by Oct 02 2026 11:59PM**.**  If you will need more time than this to complete your revisions, please reply to this message or contact the journal office at plosone@plos.org. When you're ready to submit your revision, log on to https://www.editorialmanager.com/pone/ and select the 'Submissions Needing Revision' folder to locate your manuscript file.

- A letter that responds to each point raised by the academic editor and reviewer(s). You should upload this letter as a separate file labeled 'Response to Reviewers'.
- A marked-up copy of your manuscript that highlights changes made to the original version. You should upload this as a separate file labeled 'Revised Manuscript with Track Changes'.
- An unmarked version of your revised paper without tracked changes. You should upload this as a separate file labeled 'Manuscript'.

If you would like to make changes to your financial disclosure, please include your updated statement in your cover letter. Guidelines for resubmitting your figure files are available below the reviewer comments at the end of this letter.

If applicable, we recommend that you deposit your laboratory protocols in protocols.io to enhance the reproducibility of your results. Protocols.io assigns your protocol its own identifier (DOI) so that it can be cited independently in the future. For instructions see: https://journals.plos.org/plosone/s/submission-guidelines#loc-laboratory-protocols. Additionally, PLOS One offers an option for publishing peer-reviewed Lab Protocol articles, which describe protocols hosted on protocols.io. Read more information on sharing protocols at https://plos.org/protocols?utm_medium=editorial-email&utm_source=authorletters&utm_campaign=protocols.

As the corresponding author, your ORCID iD is verified in the submission system and will appear in the published article. PLOS supports the use of ORCID, and we encourage all coauthors to register for an ORCID iD and use it as well. Please encourage your coauthors to verify their ORCID iD within the submission system before final acceptance, as unverified ORCID iDs will not appear in the published article. *Only*  the individual author can complete the verification step; PLOS staff *cannot*  verify ORCID iDs on behalf of authors.

We look forward to receiving your revised manuscript.

Kind regards,

Kahsu Gebrekidan, Ph.D.

Academic Editor

PLOS One

Journal Requirements:

If the reviewer comments include a recommendation to cite specific previously published works, please review and evaluate these publications to determine whether they are relevant and should be cited. There is no requirement to cite these works unless the editor has indicated otherwise.

Please review your reference list to ensure that it is complete and correct. If you have cited papers that have been retracted, please include the rationale for doing so in the manuscript text, or remove these references and replace them with relevant current references. Any changes to the reference list should be mentioned in the rebuttal letter that accompanies your revised manuscript. If you need to cite a retracted article, indicate the article’s retracted status in the References list and also include a citation and full reference for the retraction notice.

[Note: HTML markup is below. Please do not edit.]

Reviewers' comments:

Reviewer's Responses to Questions

**Comments to the Author**

Reviewer #2: (No Response)

**********

2. Is the manuscript technically sound, and do the data support the conclusions?

Reviewer #2: Yes

**********

3. Has the statistical analysis been performed appropriately and rigorously?

Reviewer #2: Yes

**********

4. Have the authors made all data underlying the findings in their manuscript fully available?

The PLOS Data policy

Reviewer #2: Yes

**********

5. Is the manuscript presented in an intelligible fashion and written in standard English?

Reviewer #2: Yes

**********

Reviewer #2: Thank you for revising the manuscript and addressing most of my previous comments. I appreciate the authors' efforts to improve the manuscript. However, one methodological issue remains unresolved and requires clarification before the manuscript can be considered for publication.

Comment on the Sampling Procedure

The explanation provided regarding the sampling procedure remains mathematically inconsistent. The manuscript indicates that the total sampling frame was N = 422 and the required sample size was n = 305, giving a sampling interval of:

K = N/n = 422/305 ≈ 1.38.

The authors now state that they used every 2nd interval, but this is not supported by the reported figures. Selecting every second eligible participant from a population of 422 would yield approximately 211 participants, not the reported sample size of 305.

Therefore, the current description of the sampling method remains unclear.

The authors should either:

1. Provide the correct sampling frame and calculations if systematic random sampling was indeed employed; or

2. Revise the methodology to accurately reflect the actual recruitment procedure. If nearly every eligible child admitted during the study period was enrolled until the required sample size was reached, the appropriate description would be consecutive recruitment (or consecutive sampling) rather than systematic random sampling.

Please ensure that the sampling method described is mathematically consistent with the reported sample size and sampling frame.

**********

what does this mean?). If published, this will include your full peer review and any attached files.

If you choose “no”, your identity will remain anonymous but your review may still be made public.

**Do you want your identity to be public for this peer review?**  For information about this choice, including consent withdrawal, please see our Privacy Policy

Reviewer #2: No

**********

[NOTE: If reviewer comments were submitted as an attachment file, they will be attached to this email and accessible via the submission site. Please log into your account, locate the manuscript record, and check for the action link "View Attachments". If this link does not appear, there are no attachment files.]

To ensure your figures meet our technical requirements, please review our figure guidelines: https://journals.plos.org/plosone/s/figures

You may also use PLOS’s free figure tool, NAAS, to help you prepare publication quality figures: https://journals.plos.org/plosone/s/figures#loc-tools-for-figure-preparation.

NAAS will assess whether your figures meet our technical requirements by comparing each figure against our figure specifications.

Attachment Submitted filename: reviewer.docx

# Author comment: Author response to Decision Letter 2

**DOI:** 10.1371/journal.pone.0358705.r004

26 Aug 2026

Reviewer #2: Thank you for revising the manuscript and addressing most of my previous comments. I appreciate the authors' efforts to improve the manuscript. However, one methodological issue remains unresolved and requires clarification before the manuscript can be considered for publication.

Comment on the Sampling Procedure

The explanation provided regarding the sampling procedure remains mathematically inconsistent. The manuscript indicates that the total sampling frame was N = 424 and the required sample size was n = 305, giving a sampling interval of:

K = N/n = 422/305 ≈ 1.38.

The authors now state that they used every 2nd interval, but this is not supported by the reported figures. Selecting every second eligible participant from a population of 422 would yield approximately 211 participants, not the reported sample size of 305.

Therefore, the current description of the sampling method remains unclear.

The authors should either:

1. Provide the correct sampling frame and calculations if systematic random sampling was indeed employed; or

2. Revise the methodology to accurately reflect the actual recruitment procedure. If nearly every eligible child admitted during the study period was enrolled until the required sample size was reached, the appropriate description would be consecutive recruitment (or consecutive sampling) rather than systematic random sampling.

Please ensure that the sampling method described is mathematically consistent with the reported sample size and sampling frame.

Answer:“The required sample size was calculated using the Cox formula and was determined to be 305 participants. During the study period, all eligible children who met the predefined inclusion and exclusion criteria were recruited consecutively as they were admitted. Recruitment continued until the required sample size of 305 participants was reached. Therefore, consecutive sampling (consecutive recruitment) was used.”

Attachment Submitted filename: Response_to_Reviewers_auresp_2.docx

# Peer review: Decision Letter 2

**DOI:** 10.1371/journal.pone.0358705.r005

**Authors:** Kahsu Gebrekidan

3 Sep 2026

Time to Recovery and Its Predictors Among Pediatric Patients with Severe Community-Acquired Pneumonia Admitted to Public General Hospitals in Tigray, Ethiopia, 2025: A Prospective Follow-Up Study

PONE-D-25-63360R2

Dear Dr. Tekle,

We’re pleased to inform you that your manuscript has been judged scientifically suitable for publication and will be formally accepted for publication once it meets all outstanding technical requirements.

Within one week, you’ll receive an e-mail detailing the required amendments. When these have been addressed, you’ll receive a formal acceptance letter and your manuscript will be scheduled for publication.

An invoice will be generated when your article is formally accepted. Please note, if your institution has a publishing partnership with PLOS and your article meets the relevant criteria, all or part of your publication costs will be covered. Please make sure your user information is up-to-date by logging into Editorial Manager at Editorial Manager® and clicking the ‘Update My Information' link at the top of the page. For questions related to billing, please contact billing support.

If your institution or institutions have a press office, please notify them about your upcoming paper to help maximize its impact. If they’ll be preparing press materials, please inform our press team as soon as possible -- no later than 48 hours after receiving the formal acceptance. Your manuscript will remain under strict press embargo until 2 pm Eastern Time on the date of publication. For more information, please contact onepress@plos.org.

Kind regards,

Kahsu Gebrekidan, Ph.D.

Academic Editor

PLOS One

Additional Editor Comments (optional):

Reviewers' comments:

Reviewer's Responses to Questions

**Comments to the Author**

Reviewer #2: All comments have been addressed

**********

2. Is the manuscript technically sound, and do the data support the conclusions?

Reviewer #2: Yes

**********

3. Has the statistical analysis been performed appropriately and rigorously?

Reviewer #2: Yes

**********

4. Have the authors made all data underlying the findings in their manuscript fully available?

The PLOS Data policy

Reviewer #2: Yes

**********

5. Is the manuscript presented in an intelligible fashion and written in standard English?

Reviewer #2: Yes

**********

Reviewer #2: Thank you for the clarification. The authors have adequately addressed my concern regarding the inconsistency in the sampling procedure. The sampling method has been appropriately revised from systematic random sampling to consecutive sampling, consistent with the actual recruitment procedure and the reported sample size. The methodological concern is therefore satisfactorily resolved.

**********

what does this mean?). If published, this will include your full peer review and any attached files.

If you choose “no”, your identity will remain anonymous but your review may still be made public.

**Do you want your identity to be public for this peer review?**  For information about this choice, including consent withdrawal, please see our Privacy Policy

Reviewer #2: No

**********

# Editor report: Acceptance letter

**DOI:** 10.1371/journal.pone.0358705.r006

**Authors:** Kahsu Gebrekidan

PONE-D-25-63360R2

PLOS One

Dear Dr. Ygzaw,

I'm pleased to inform you that your manuscript has been deemed suitable for publication in PLOS One. Congratulations! Your manuscript is now being handed over to our production team.

At this stage, our production department will prepare your paper for publication. This includes ensuring the following:

* All references, tables, and figures are properly cited

* All relevant supporting information is included in the manuscript submission,

* There are no issues that prevent the paper from being properly typeset

You will receive further instructions from the production team, including instructions on how to review your proof when it is ready. Please keep in mind that we are working through a large volume of accepted articles, so please give us a few days to review your paper and let you know the next and final steps.

Lastly, if your institution or institutions have a press office, please let them know about your upcoming paper now to help maximize its impact. If they'll be preparing press materials, please inform our press team within the next 48 hours. Your manuscript will remain under strict press embargo until 2 pm Eastern Time on the date of publication. For more information, please contact onepress@plos.org.

You will receive an invoice from PLOS for your publication fee after your manuscript has reached the completed accept phase. If you receive an email requesting payment before acceptance or for any other service, this may be a phishing scheme. Learn how to identify phishing emails and protect your accounts at https://explore.plos.org/phishing.

If we can help with anything else, please email us at customercare@plos.org.

Thank you for submitting your work to PLOS One and supporting open access.

Kind regards,

PLOS One Editorial Office Staff

on behalf of

Dr. Kahsu Gebrekidan

Academic Editor

PLOS One
